# Bimetallic Cobalt–Copper Nanoparticle-Decorated Hollow Carbon Nanofibers for Efficient CO_2_ Electroreduction

**DOI:** 10.3389/fchem.2022.904241

**Published:** 2022-04-29

**Authors:** Congyi He, Siyu Wang, Xingxing Jiang, Qi Hu, Hengpan Yang, Chuanxin He

**Affiliations:** College of Chemistry and Environmental Engineering, Shenzhen University, Shenzhen, China

**Keywords:** bimetallic catalysts, copper–cobalt bimetal, carbon nanofibers, CO_2_ reduction, Electrocatalysis

## Abstract

Bimetallic materials are one of the most promising catalysts for the electrochemical reduction of CO_2_, but there are still many challenges to be overcome on the route to industrialization. Herein, a series of carbon nanofiber-supported bimetallic cobalt–copper catalysts (Co_x_Cu_y_/CFs) are designed and constructed through the electrospinning technique and a subsequent pyrolysis procedure. Small-sized Co–Cu nanoparticles are homogenously distributed on the porous carbon nanofibers, which can significantly improve the utilization rate of metal sites and greatly reduce the loading amount of metals. Moreover, different product distributions and catalytic performance can be obtained in CO_2_ reduction *via* adjusting the metal proportion of Co_x_Cu_y_/CFs. Especially, Co_3_Cu/CFs can bring forth a 97% total faradaic efficiency (FE) of CO (68%) and HCOOH (29%) at –0.8 V_RHE_ cathode potential in 0.5 M KHCO_3_ electrolyte. Furthermore, the hierarchical pores can firmly confine the small Co–Cu nanoparticles and keep them from easy agglomeration during electrolysis, eventually leading to 60 h of stability for Co_3_Cu/CFs in CO_2_ electroreduction. This study might provide a facile and economic method to fabricate efficient bimetallic catalysts for CO_2_ electroreduction and other electrocatalysis applications.

## Introduction

CO_2_ electroreduction can convert greenhouse gas CO_2_ into renewable fuels and industrial building-block chemicals, which has been advocated as a promising candidate for the artificial carbon cycle (Qiao et al., 2014; Sharifian et al., 2021; Zhang et al., 2021). Electrochemical reduction of CO_2_ can be motivated by vast amounts of excess electricity from renewable energy resources, for example, wind, tide, and solar power plants (Benson et al., 2009; Fu et al., 2019). However, the CO_2_ molecule has a very high chemical stability; hence, appropriate catalysts are needed to activate them (Hori et al., 2008; Appel et al., 2013).

Therefore, a series of catalysts have been constructed to enhance the efficiency of CO_2_ electroreduction, including molecular catalysts (Appel and Helm, 2014; Nichols and Machan, 2019; and Bonin et al., 2017), heterodoped carbon catalysts (Sun, 2021; Kumar et al., 2013), oxide-derived catalysts (Duan et al., 2021; Duan et al., 2018), single-atom catalysts (Yang et al., 2019; Chen Jia et al., 2021; Wei et al., 2022; and Zhang et al., 2022), and multimetallic catalysts (Lin Jia et al., 2021; Vasileff et al., 2018; and Jia et al., 2022). Among these available catalysts, bimetallic catalysts have exhibited remarkable performance in CO_2_ reduction. Bimetallic materials not only change the electronic structures of the single component (Yang et al., 2021) but also create new active sites to regulate the binding energy of key intermediates during CO_2_ reduction (Jeoung and Dobbek, 2007; Yu et al., 2021; Cheng et al., 2021). Meanwhile, carbon materials (e.g., carbon black, graphite powder, and carbon nanotubes) are utilized as supports or carriers for bimetallic catalysts in actual electrolysis, which can improve the dispersion and conductivity (Jia et al., 2022). However, these carbon materials need pretreatment, including purification or surface functionalization, which might damage the porous structure and electrical conductivity (Liu et al., 2014). Therefore, it is still urgent to design a simple and effective approach to carbon supported with excellent activity in CO_2_ electroreduction.

In this study, we report the facile synthesis of several Co–Cu bimetallic catalysts, that is, Co–Cu bimetallic nanoparticles/porous carbon nanofiber (Co_x_Cu_y_/CF) composites using the electrospinning technique and thermal treatment. In the composites, Co_x_Cu_y_ nanoparticles are uniformly and stably dispersed on the abundant poles of carbon nanofibers, rather than simply being absorbed or drop-coated on the surface. This structure can largely expose the Co_x_Cu_y_ nanoparticles onto the reaction interface of CO_2_ electroreduction and greatly improve the efficiency of electronic transmission. Furthermore, we also systematically investigated the effect of the mole ratio of Co and Cu components on the product distribution and faradaic efficiency. The results indicated that the Co_3_Cu/CF catalyst with a mole ratio of 3:1 displayed an outstanding catalytic activity and long-term stability in CO_2_ electroreduction.

## Materials and Methods

### Chemicals and Characterizations

All reagents were used as received without further purification.

Electrochemical tests were performed with a CHI 760e electrochemical Station (Shanghai Chenhua Instruments Company). Gaseous products were detected by gas chromatography (Shimadzu, GC-2014c) with a flame ionization detector (FID) and a thermal conductivity detector (TCD). Liquid products were detected using a nuclear magnetic resonance spectrometer (NMR, Ascend 400, Bruker, Germany). The micromorphology, crystalline structure, and element mapping were obtained by a field emission scanning electron microscope (FE-SEM, FEI JEOL-7800F) and a high-resolution transmission electron microscope (HR-TEM, JEM-2100F). The metal amount in the as-synthesized catalyst was detected using inductively coupled plasma-optical emission spectrometry (ICP-OES, OPTIMA2100DV). N_2_ adsorption/desorption curves were achieved by a specific surface and porosity analyzer (Micromeritics ASAP 2460) and calculated using the Brunauer–Emmett–Teller (BET) equation. X-ray diffraction (XRD) patterns were recorded with an X-ray powder diffractometer (Rigaku MiniFlex 600) with Cu Kα radiation (k = 1.5406 Å). Raman spectra were acquired with a laser Raman spectrometer (LabRAM HR Evolution, HORBIA FRANCE SAS) with a 633-nm laser excitation. X-ray photoelectron spectra (XPS) were recorded on an X-ray photoelectron spectrometer (ThermoVG Scientific ESCALAB 250) with Al Kα X-ray as the source.

### Synthesis of Catalysts

All the five samples in this study were prepared by electrospinning technology. The preparation steps are as follows: 7 ml of N, N-dimethylformamide, 0.5 g polyacrylonitrile (PAN), and 0.75 g ZIF-8 nanoparticles were put into a beaker and stirred until they were evenly mixed into a white viscous solution. Then, 0.2183 g of Co(NO_3_)_2_·6H_2_O (0.00075 mol) and 0.061 g of Cu(NO_3_)_2_·3H_2_O (0.00025 mol) were added, and stirring was continued for at least 20 h or until the mixture was fully mixed to obtain a purple viscous spinning precursor solution. This precursor solution was injected into the syringe and electrospun to polymer fibers. After spinning, the polymer fibers were put into vacuum drying oven at 60°C for at least 12 h, and the dried polymer fiber was pre-oxidized in a muffle furnace. Then those pre-oxidized fibers were carbonized in nitrogen atmosphere. The initial temperature was set at 25°C, raised to 900°C at the rate of 5°C/min, and maintained for another 2 h. The as-synthesized catalyst was named as Co_3_Cu/CFs.

Another four catalysts with different metal ratios can be obtained by changing the molar ratio of metal precursors Co (NO_3_)_2_·6H_2_O and Cu (NO_3_)_2_·3H_2_O, including 1/0, 1/1, 1/3, and 0/1. The as-prepared samples were named as Co/CFs, CoCu/CFs, CoCu_3_/CFs, and Cu/CFs.

### Electrochemical Measurements

All the five catalysts were powdered and drop-coated onto a carbon paper (SGL Carbon Corporate) to get a useful working electrode. CO_2_ reduction activity was tested in a typical H-type electrochemical cell separated by an anion exchange membrane between anodic and cathodic chambers, with a Pt foil as the counter electrode and an Ag/AgCl as the reference electrode; 0.5 M KHCO_3_ solution was employed as the electrolyte and bubbled with high purity CO_2_ or N_2_ (99.995%). The original potentials measured in this manuscript were converted to the reversible hydrogen electrode (RHE) *via* the Nernst equation:

E(RHE)=E(Ag/AgCl)+0.199+0.059×pH. (1)

Products from CO_2_ reduction were analyzed at various cathodic potentials with a fixed time of 15 min, and the gaseous components were directly injected into gas chromatography. The liquid-phase products were detected *via* ^1^H NMR spectra. The Faraday efficiencies of the products were calculated *via* the following equations. Q is the total charge transferred through the working electrode at different potentials; m is the number of electrons transferred, which is 2 for HCOOH, CO, and H_2_, and 8 for CH_4_; n is the mole numbers of products; and F is the Faradaic constant (96,485 C mol^−1^).

$$\mathrm{FE}=\frac{Q_{\mathrm{product}}}{Q_{\mathrm{total}}}=\frac{m\times n\times F}{Q_{\mathrm{total}}}$$ (2)

## Results and Discussions

### Characterizations of Catalysts

**FIGURE 1:**
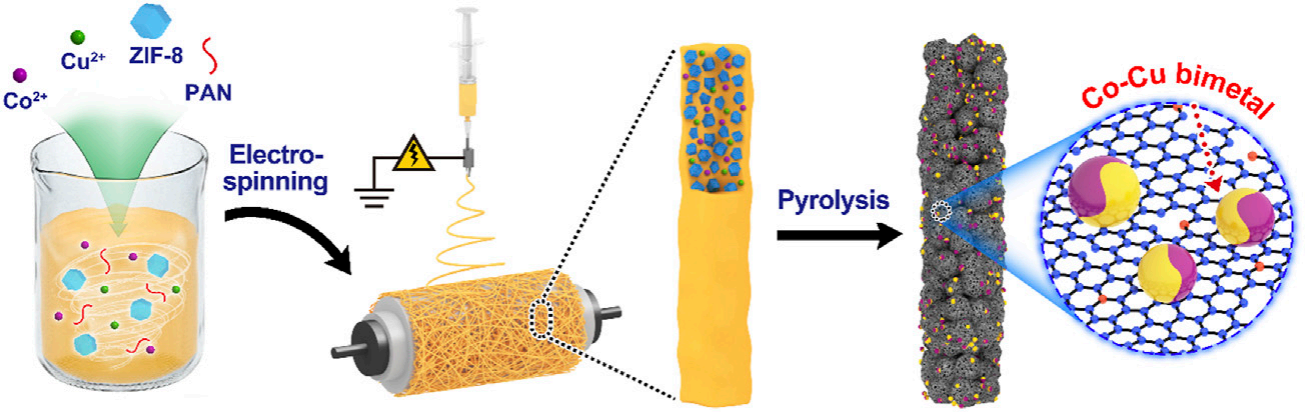
Synthesis strategy of Co–Cu bimetallic nanoparticle-decorated carbon nanofibers.

The specific preparation process of the material is described in Figure 1. First, Co(NO_3_)_2_·6H_2_O, Cu(NO_3_)_2_·3H_2_O, ZIF-8 nanoparticles as well as PAN were dissolved in DMF to prepare a precursor solution, and then an electrospinning technology was used under constant conditions to get PAN nanofibers with different Co/Cu mole ratios. Then, the Co–Cu/PAN nanofibers were heated to 900°C under a N_2_ atmosphere for carbonization. Notably, there are no extra surfactants or reductants involved in the whole procedure. The polymer linkers were pyrolyzed and carbonized to generate the main body of carbon nanofibers, and ZIF-8 nanoparticles collapsed to form the abundant mesopores and macropores through these nanofibers (Yang et al., 2020a). Co^2+^ and Cu^2+^ ions were reduced by organic linkers, and the bimetallic nanoparticles were engendered with a smaller particle size due to the confinement of the polymer and ZIF-8 nanoparticles. The compositions of these bimetallic nanoparticles were tuned by the feeding ratio of Co(NO_3_)_2_·6H_2_O and Cu(NO_3_)_2_·3H_2_O precursors, eventually generating Co_3_Cu/CFs, Co/CFs, CoCu/CFs, CoCu_3_/CFs, and Cu/CFs.

**FIGURE 2:**
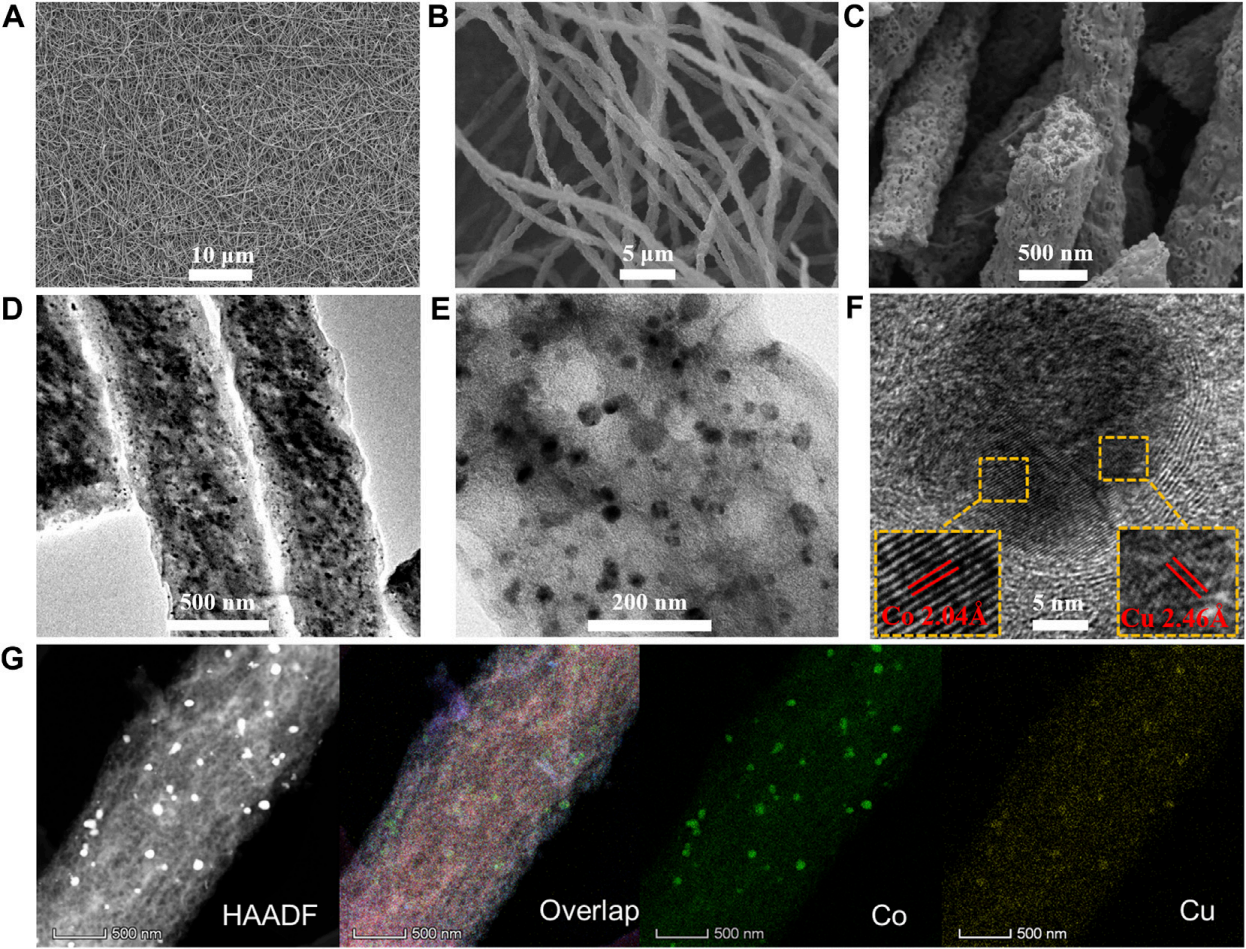
**(A–C)** SEM and **(D–E)** TEM images with different resolutions of Co_3_Cu/CFs, respectively; **(F)** HR-TEM images of Co_3_Cu/CFs: inset shows the lattice fringes of Co and Cu; **(G)** HAADF-STEM and elemental mapping images of Co_3_Cu/CFs.

**FIGURE 3:**
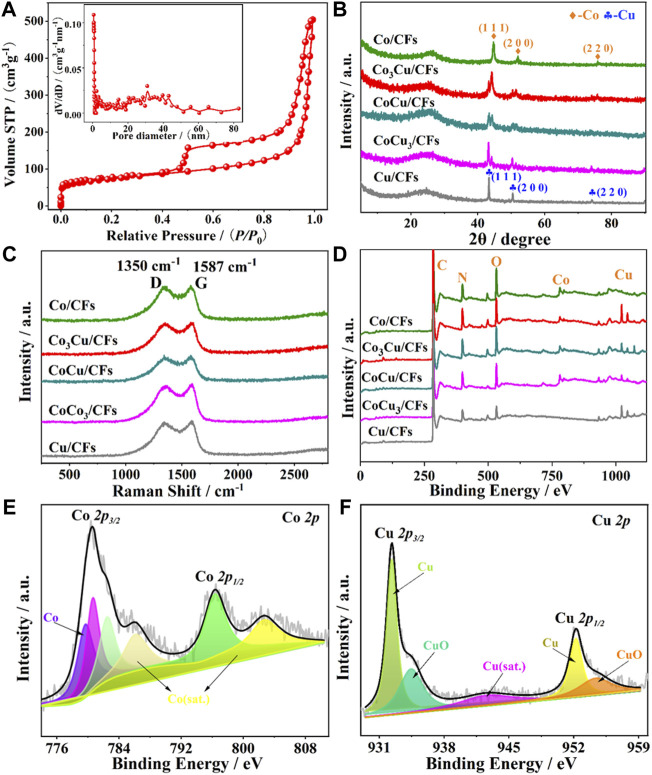
**(A)** N_2_ sorption isotherms of Co_3_Cu/CFs: inset displays the pore size distribution; **(B)** XRD patterns, **(C)** Raman spectra, and **(D)** XPS survey spectra of the five catalysts; **(E)** Co *2p* and **(F)** Cu *2p* fine XPS spectra of Co_3_Cu/CFs.

As shown in Figure 2, the surface morphology and nanostructure of the as-synthesized Co_3_Cu/CF catalyst were recorded by FE-SEM and HR-TEM, respectively. The diameter of the carbon nanofibers in Co_3_Cu/CFs ranges from 500 to 600 nm. The length is in the scale of hundreds of micrometers, and the interlaced nanofibers furtherly form a network structure (Figures 2A,B). Moreover, abundant hollow pores, in the size range of dozens of nanometers, could be easily seen throughout Co_3_Cu/CFs (Figures 2C,D). N_2_ sorption isotherms (Figure 3A) further demonstrate that Co_3_Cu/CFs have type Ⅳ sorption isotherm, belonging to the mesoporous structure. Co–Cu nanoparticles, in an ∼20 nm diameter range, are evenly immobilized within the hollow pores of carbon nanofibers (Figures 2D,E).

In addition, Figure 2F shows the clear HR-TEM image of an independent Co–Cu bimetallic nanoparticle, and the interplanar spacing of crystalline lattices marked with red lines is measured as 2.04 Å and 2.46 Å, corresponding to the Co (111) and Cu (111) planes (Liu et al., 2014; Kim et al., 2017), respectively. Furthermore, the high-angle annular dark field STEM (HAADF-STEM) and elemental mapping images indicate that Co_3_Cu/CFs contain Co and Cu elements. The good match between the emerging positions of Co and Cu elements directly proves the formation of Co–Cu bimetallic nanoparticles. According to the SEM, HR-TEM (Supplementary Figure S1,S2), and N_2_ sorption isotherms (Supplementary Figure S3, Supplementary Table S1), Co/CFs, CoCu/CFs, CoCu_3_/CFs, and Cu/CFs display similar interlaced nanofibers, abundant hollow pores, and uniform nanoparticles. Hence, the regulation of metal ratio would not significantly change the surface morphology and nanostructure. The actual Co/Cu ratios in the as-synthesized catalyst were detected by ICP-OES. CoCu/CFs, Co_3_Cu/CFs, and CoCu_3_/CFs own Co/Cu ratios of 1/0.95, 3/0.96, and 0.98/3, respectively, which are close to the original ratios in precursor solutions.

As shown in the XRD patterns (Figure 3B), the sharp diffraction peaks at 44.9°, 52.5°, and 76.2° can be seen in Co/CF samples, which are attributed to the Co (111), (200), and (220) planes (JCPDS 89-7093), respectively. Those peaks at 43.3°, 50.5°, and 74.2° of the Cu/CF samples are in accordance with the Cu (111), (200), and (220) planes (JCPDS 89-7093), respectively. The XRD patterns of Co_3_Cu/CFs, CoCu/CFs, and CoCu_3_/CFs possess both Co and Cu diffraction peaks, further verifying the formation of Co–Cu bimetallic nanoparticles. The Raman spectra (Figure 3C) of the five samples contain characteristic peaks around 1,350 cm^−1^ and 1,578 cm^−1^, related to the d band of defective carbon and the G band of graphite carbon (Kumar et al., 2013), respectively. Co_3_Cu/CFs have the largest intensity ratio of D and G bands (I_D_/I_G_, 1.01), indicating more defects and more potential active sites. The survey XPS spectra of Co_3_Cu/CFs (Figure 3D, Supplementary Figure S4) confirm the presence of Co, Cu, C, N, and O elements. The high-resolution Co *2p* XPS spectrum of Co_3_Cu/CFs (Figure 3E) exhibits four peaks, including Co *2p*_*3/2*_ (780.3 eV), Co *2p*_*1/2*_ (795.6 eV), and two Co satellite peaks. Two strong peaks in the Cu *2p* XPS spectrum (Figure 2F) that appear at 932.3 and 952.0 eV can be indexed to Cu *2p*_*3/2*_ and Cu *2p*_*1/2*_, respectively. Notably, oxide peaks can be detected in Co and Cu *2p* XPS spectra of Co_3_Cu/CFs because of the easy oxidation of metallic Co and Cu nanoparticles (Xie et al., 2021; Kim et al., 2017). Similar C, N, Co, or Cu species could also be observed in the XPS spectra of another four samples (Supplementary Figure S5–S8).

### CO_2_ Electroreduction Tests

**FIGURE 4:**
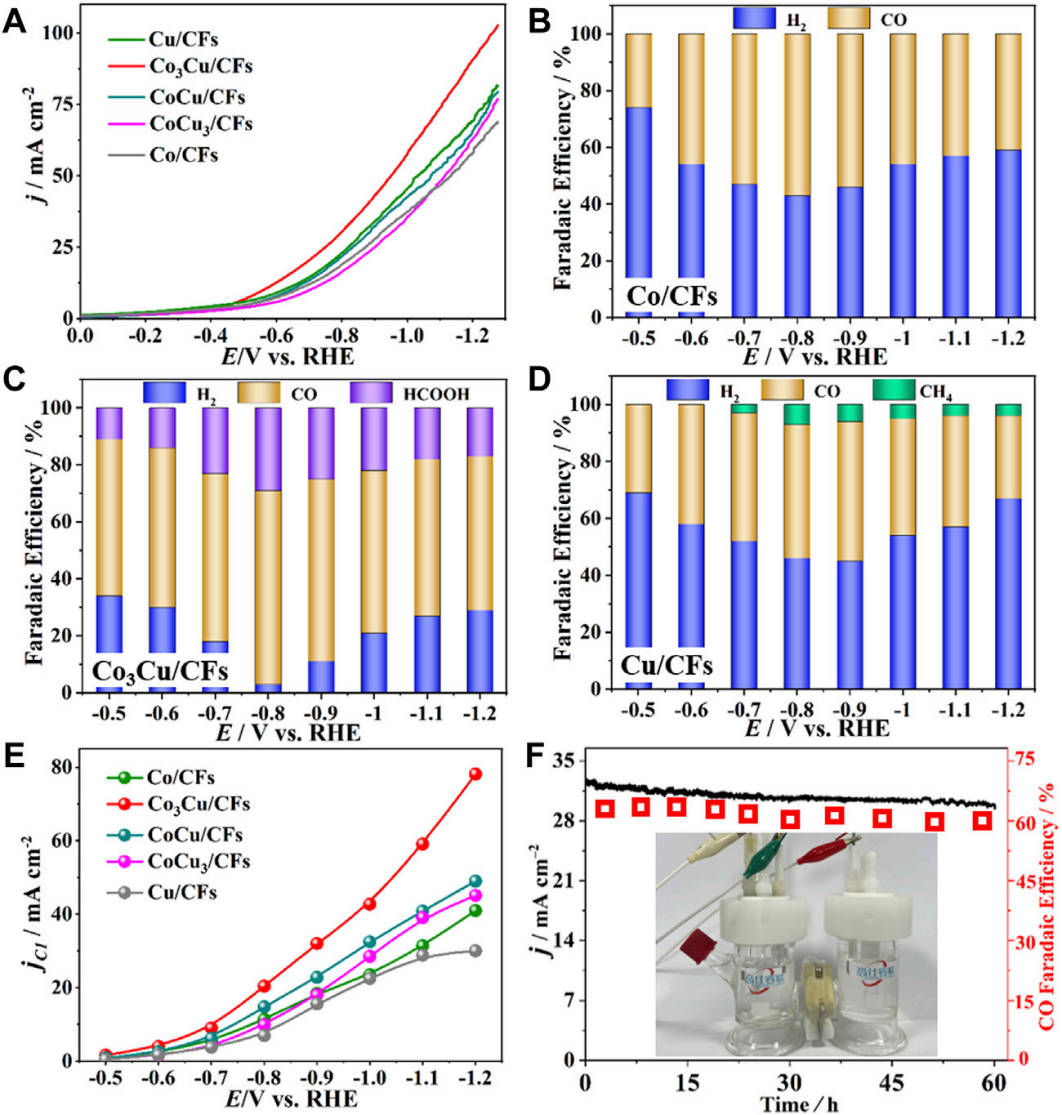
**(A)** LSV curves of the five samples recorded in a CO_2_-saturated 0.5 M KHCO_3_ electrolyte; Faradaic efficiencies of **(B)** Co/CFs, **(C)** Co_3_Cu/CFs, and **(D)** Cu/CFs in a 0.5 M KHCO_3_ electrolyte; **(E)** C1 product partial current densities of the five samples; **(F)** long-term tests of Co_3_Cu/CFs at −0.8 V_RHE_ in a 0.5 M KHCO_3_ electrolyte.

The CO_2_ electroreduction performances of Co–Cu bimetallic catalysts were investigated and compared with the performance of pure Co or Cu catalysts in a typical H-type electrochemical cell. All the catalysts were pre-activated using cyclic voltammetry until stable profiles were obtained. Figure 4A and Supplementary Figure S9 present the linear sweep voltammetry (LSV) curves of five samples in N_2_-saturated or CO_2_-saturated 0.5 M KHCO_3_. The cathodic current densities of all five catalysts were measured to be approximately doubled in CO_2_ than those in the N_2_-saturated electrolyte, indicating the potential catalytic activity in CO_2_ reduction (Zhang et al., 2014). In addition, LSV tests also prove that Co_3_Cu/CFs show the highest current density among the five catalysts (Figure 4A).

In order to further evaluate the catalytic activities and quantify the product distribution from CO_2_ electroreduction, potential dependent CO_2_ electrolysis using five catalysts was conducted in a 0.5 M KHCO_3_ electrolyte from –0.5 V_RHE_ to –1.2 V_RHE_ applied cathode potential. Gas products were directly injected into gas chromatography for on-line analysis, and liquid products in the catholyte were detected using ^1^H NMR after each electrolysis. The product distribution and faradaic efficiencies (FEs) are summarized in Figure 4 and Supplementary Figure S10. For comparison of the electrocatalytic activities, Co/CFs and Cu/CFs with pure Co and Cu nanoparticles were also synthesized and assessed in CO_2_ electrolysis. As shown in Figure 4B, Co/CFs generated CO as the only product from the CO_2_ reduction and H_2_ from the hydrogen evolution reaction (HER), with a 57% maximum FE for CO at –0.8 V_RHE_ applied potential. As for Cu/CFs (Figure 4D), it produced H_2_, CO, and CH_4_ during electrolysis, with a 49% maximum FE for CO at –0.9 V_RHE_. CH_4_ is one of the urgent products with the transfer of eight electrons (Sharifian et al., 2021); however, highest FEs of CH_4_ only reach 7% at –0.8 V_RHE_. Both Co/CFs and Cu/CFs show certain activities in CO_2_ electroreduction, but neither of them could effectively suppress the HER process, and the FEs of H_2_ range from 43 to 74%. As displayed in Figure 4C and Supplementary Figure S10, doping Co with Cu causes a significant increment in CO_2_ catalysis. Compared to Co/CFs and Cu/CFs, the CO_2_ reduction procedure on Co_3_Cu/CFs, CoCu/CFs, and CoCu_3_/CFs became much more dominant than the HER procedure. In particular, the maximum FE for C1 production (HCOOH and CO) increases to 97% at –0.8 V_RHE_ using the Co_3_Cu/CFs catalyst, and the HER is totally suppressed to only a 3% FE of H_2_. CoCu/CFs and CoCu_3_/CFs have a similar tendency of FEs for CO_2_ electrolysis as that of Co_3_Cu/CFs, but they acquire lower total FEs of CO and HCOOH throughout the applied potential.

Chronoamperometry (CA) was used to evaluate the total current density during CO_2_ electrolysis, and five samples achieved very close total current densities from –0.5 V_RHE_ to –1.2 V_RHE_ (Supplementary Figure S11). In addition, the partial current densities (*j*_*C1*_) for C1 products (CO, HCOOH, and CH_4_) were normalized by the total current densities and FEs at each cathode potential. Co_3_Cu/CFs brought forth a significantly higher partial current density than Co/CFs, Cu/CFs, CoCu/CFs, and CoCu_3_/CFs within the potential range, and got a maximum *j*_*C1*_ of 78.1 mA cm^−2^ at –1.2 V_RHE_ (Figure 4E). Therefore, Co_3_Cu/CFs possess remarkable catalytic activity in CO_2_ reduction, and they can also successfully suppress the HER procedure at relatively high cathode potentials. Moreover, it is extremely important to estimate the long-term durability of bimetallic catalysts because increasing the applied potentials and heavy current densities might seriously impact the structural stability (Vasileff et al., 2018; Jia et al., 2022). Long-term tests of potentiostatic CO_2_ electrolysis were conducted using Co_3_Cu/CFs catalysts at –0.8 V_RHE_ cathode potential where the best FEs of C1 products were obtained. The gaseous products were detected on-line every 6 h, and the corresponding CO FEs and current densities *versus* time are plotted in Figure 4F. Both CO FEs and partial current densities of the Co_3_Cu/CF catalyst exhibited only small declines during the 60 h electrolysis, retaining approximately 90% of the original values and manifesting excellent stability in CO_2_ electroreduction. Co_3_Cu/CFs were characterized after a long-term electrolysis by TEM (Supplementary Figure S12), and the small Co_3_Cu/CFs did not agglomerate together. As described previously (Figures 2D,E), the Co–Cu bimetallic nanoparticles are uniformly dispersed and firmly confined within the hierarchical pores of carbon nanofibers, and separated from easy agglomeration during electrolysis.

**FIGURE 5:**
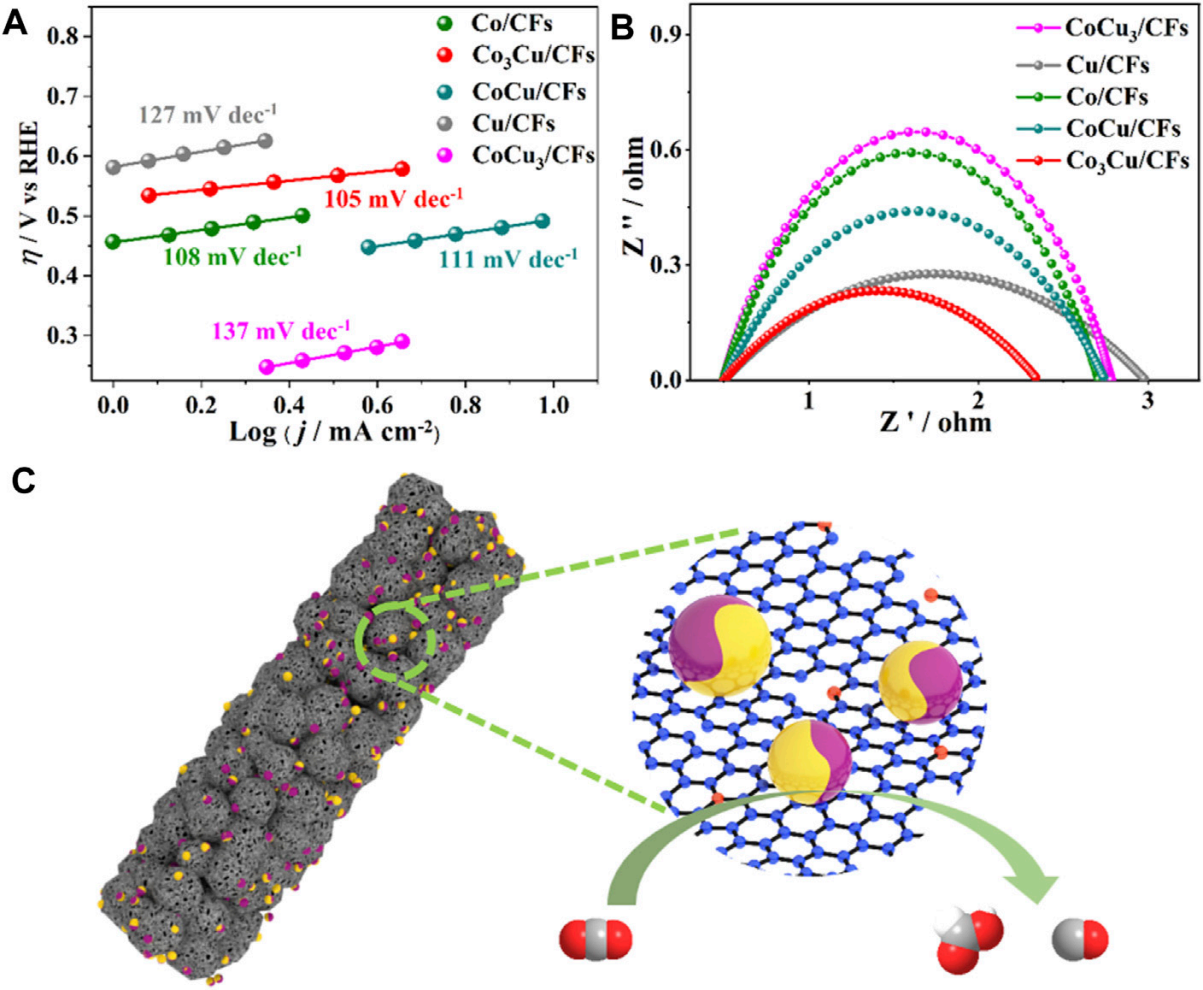
**(A)** Tafel plots of five samples during the CO_2_ electroreduction process; **(B)** EIS Nyquist spectra of the five samples; **(C)** catalytic mechanism of Co_3_Cu/CFs for the reduction of CO_2_ to CO and HCOOH.

The aforementioned experimental results demonstrate the outstanding activities of Co_3_Cu/CFs in CO_2_ reduction. The mechanism of the high performance was first investigated using Tafel slopes within sufficiently low overpotential ranges. As presented in Figure 5A, 105 mV dec^−1^ Tafel value is observed on Co_3_Cu/CFs, confirming the first electron transfer from CO_2_ to CO_2_•^−^ as the rate determining step (Chen Jia et al., 2021). Compared to those of Co/CFs (108 mV dec^−1^), Cu/CFs (127 mV dec^−1^), CoCu/CFs (111 mV dec^−1^), and CoCu_3_/CFs (137 mV dec^−1^), the lower Tafel value of Co_3_Cu/CFs indicates faster reaction kinetics in CO_2_ reduction. As mentioned previously, four electron product CH_4_ was only obtained with the Cu/CF catalyst, and the bimetallic Co_x_Cu_y_/CFs could produce CO and HCOOH. Metallic Cu owns relatively strong binding energies of *COOH and *CO intermediates compared to pure metallic Co, and these intermediates could be stabilized and further reduced to hydrocarbons or alcohols (Duan et al., 2018; Mun et al., 2019). In Co_x_Cu_y_/CF samples, the catalytic behavior of metallic Cu was totally altered *via* fusing it with Co composition within the same nanoparticles. The binding energies of *COOH and *CO intermediates were weakened enough to be released from the catalyst surface, increasing the tendency toward HCOOH and CO production.

The electrochemical active surface area and the conductivity property of these five samples were also measured *via* double-layer capacitance (C_dl_) and electrochemical impedance spectroscopy (EIS). Compared with Co/CFs (31.5 mF cm^−2^), Cu/CFs (17.5 mF cm^−2^), CoCu/CFs (30.0 mF cm^−2^), and CoCu_3_/CFs (21.5 mF cm^−2^), Co_3_Cu/CFs have a much higher C_dl_ value of 45.5 mF cm^−2^ (Supplementary Figure S13), manifesting the larger ECSA and more active sites for CO_2_ reduction (Yang et al., 2020b; Hao et al., 2022). In addition, the EIS curves in Figure 5B prove that Co_3_Cu/CFs show a relatively small impedance than those of the other samples, which is beneficial to faster electron transport as well as better conductivity (Wei et al., 2022; Liu et al., 2014). The larger ECSA and good conductivity of Co_3_Cu/CFs is consistent with its higher current densities in LSV and electrolysis tests. As illustrated in Figure 5C, Co_3_Cu/CFs possess highly graphitized and multi-level porous carbon nanofibers, which can accelerate the electron transmission and expose abundant bimetallic Co–Cu sites for CO_2_ reduction, eventually leading to the remarkable partial current densities for C1 products.

## Conclusion

In summary, an efficient Co_3_Cu/CF catalyst was created with bimetallic Co–Cu nanoparticles evenly distributed within porous carbon nanofibers, which exhibited superior catalytic activities in CO_2_ reduction. A total 97% total faradaic efficiency of CO and HCOOH could be achieved with the Co_3_Cu/CFs catalyst at –0.8 V_RHE_ cathode potential in a 0.5 M KHCO_3_ electrolyte. In addition, Co_3_Cu/CFs could also bring forth a maximum 78.1 mA cm^−2^ partial current density for C1 production and maintain 60-h of stability in long-term electrolysis. In Co_3_Cu/CFs catalysts, the doping of metallic Cu with Co can decrease the binding energies of key intermediates and increase the selectivity of CO and HCOOH. Moreover, the hierarchically porous carbon nanofibers are in favor of electron transmission and exposing active sites for CO_2_ electroreduction. Consequently, this effective strategy of composition tuning along with a tailored structure might inspire the design and preparation of robust catalysts for CO_2_ electroreduction.

## Data Availability

The original contributions presented in the study are included in the article/Supplementary Material, further inquiries can be directed to the corresponding authors.
