# Supplementary material for: Bimetallic Cobalt–Copper Nanoparticle-Decorated Hollow Carbon Nanofibers for Efficient CO2 Electroreduction
Source: Front Chem. 2022 Apr 29;10:904241. doi: 10.3389/fchem.2022.904241 (PMC9099375; doi:10.3389/fchem.2022.904241)
Supplement: Supplementary file 1 [file DataSheet1.docx]

Supplementary Material

**Bimetallic Cobalt-Copper** **Nanoparticles Decorated Hollow Carbon Nanofibers for Efficient CO_2_ Electroreduction**

Congyi He, Siyu Wang, Xingxing Jiang, Qi Hu, Hengpan Yang*, Chuanxin He*

*Shenzhen Key Laboratory for Functional Polymer, College of Chemistry and Environmental Engineering, Shenzhen University, Shenzhen, Guangdong, 518060, China*

* Corresponding Authors:

Dr. Hengpan Yang, E-mail: hpyang@szu.edu.cn

Prof. Chuanxin He, E-mail: [hecx@szu.edu.cn](mailto:hecx@szu.edu.cn)

***Contents:***

Supplementary Figures 1 to 13

Supplementary Tables 1

1. **Materials and Characterizations**

All reagents were used as received.

Linear sweep voltammograms (LSV), double-layer capacitance (C_dl_), electrochemical impedance spectroscopy (EIS) and potentiostatic electrolysis were recorded using a CHI 660C electrochemical Station (Shanghai Chenhua Instruments Company). Liquid phase products were resolved by ^1^H-NMR spectra recorded on an Ascend 400 (500 MHz, Bruker, Germany) spectrometer. Gas phase products were resolved by Gas Chromatography (Shimadzu, GC-2014c) with a flame ionization detector (FID) and a thermal conductivity detector (TCD).

The micromorphology was characterized by a field emission scanning electron microscope (FE-SEM, FEI JEOL-7800F). The crystalline structure and element mapping were obtained using transmission electron microscopy (TEM) and high-resolution TEM (HR-TEM) images and element mapping analysis using JEM-2100F field emission electron microscope. The metal amount in as-synthesized catalyst were detected by inductively coupled plasma-optical emission spectrometry (ICP-OES, OPTIMA2100DV). N_2_ adsorption/desorption curves were achieved by a specific surface and porosity analyzer (Micromeritics ASAP 2460) and calculated using the Brunauer-Emmett-Teller (BET) equation. X-ray diffraction (XRD) patterns were recorded with an X-ray powder diffractometer (Rigaku MiniFlex 600) with Cu Kα radiation (k= 1.5406 Å). Raman spectra were acquired with a laser Raman spectrometer (LabRAM HR Evolution, HORBIA FRANCE SAS) with a 633 nm laser excitation. X-ray photoelectron spectra (XPS) were recorded on an X-ray photoelectron spectrometer (ThermoVG Scientific ESCALAB 250) with Al Kα X-ray as the source.

1. **Synthesis of Catalysts and CO_2_ Reduction**
   1. ***Synthesis of five samples***

All the five samples in this manuscript were prepared by electrospinning technology. The preparation steps are as follows: 7 mL of N, N-dimethylformamide, 0.5 g polyacrylonitrile (PAN) and 0.75 g ZIF-8 nanoparticles were put into a beaker and stirred until they were evenly mixed into a white viscous solution. Then, 0.2183 g of Co(NO_3_)_2_·6H_2_O (0.00075 mol) and 0.061 g of Cu(NO_3_)_2_·3H_2_O (0.00025 mol) were added, and kept stirring for at least 20 hours until fully mixed to obtain a purple viscous spinning precursor solution. This precursor solution was injected into the syringe and electrospun to polymer fibers. After spinning, the polymer fibers were put into vacuum drying oven at 60 ℃for at least 12 hours, and the dried polymer fiber were pre-oxidized in a muffle furnace. Then those pre-oxidized fibers were carbonized in nitrogen atmosphere. The initial temperature was set at 25 ℃, raised to 900 ℃ at the rate of 5 ℃/min, and kept for another 2 hours. The as-synthesized catalyst is named as Co_3_Cu/CFs.

Another four catalysts with different metal ratio can be obtained by changing the molar ratio of metal precursors Co(NO_3_)_2_·6H_2_O and Cu(NO_3_)_2_·3H_2_O, including 1/0, 1/1, 1/3, and 0/1. The as-prepared samples were named as Co/CFs, CoCu/CFs, CoCu_3_/CFs and Cu/CFs.

- 1. ***CO_2_ Reduction Procedure***

Linear sweep voltammograms, double-layer capacitance, electrochemical impedance spectroscopy and potentiostatic electrolysis were conducted in a in typical H-type electrochemical cell separated by an anion exchange membrane between cathode and anode compartment, with a platinum mesh as the counter electrode and an Ag/AgCl as reference electrode in 0.5 M KHCO_3_ solution. The CO_2_ or N_2_ gas was A specific volume of the catalyst (Co_3_Cu/CFs, Co/CFs, CoCu/CFs, CoCu_3_/CFs and Cu/CFs) ink was then drop-casted on carbon paper (SGL Carbon Corporate) electrode to acquire an 0.5 mg cm^-2^ loading amount and then dried at room temperature. This carbon paper would be used as the working cathode for CO_2_ electrolysis. The catalyst ink was prepared via the following steps: specific amount catalyst powder (Co_3_Cu/CFs, Co/CFs, CoCu/CFs, CoCu_3_/CFs and Cu/CFs) was put into a mixture solution of 110 µL of Nafion solution (5 wt%, Dupond) and 890 µL of ethanol. The mixed solution was sonicated for 30 min to get a highly dispersed ink.

The original potentials measured in this manuscript were converted to the reversible hydrogen electrode (RHE) via the Nernst equation:

$$\boldsymbol{E}\left( \boldsymbol{RHE} \right)\boldsymbol{=E}\left( \boldsymbol{Ag/AgCl} \right)\boldsymbol{+0.199+0.059}\boldsymbol{\times}\boldsymbol{pH (1)}$$

Product from CO_2_ reduction were analysed at various cathodic potentials with a fixed time of 15 minutes, and the gaseous components were directly injected into gas chromatography. The liquid-phase products were detected via ^1^H NMR spectra. The faraday efficiencies of products were calculated via the following equation. Q is the total charge transferred through the working electrode at different potentials. m is the number of electrons transferred, which is 2 for HCOOH, CO and H_2_, 4 for CH_4_. n is the mole numbers of products, and F is the Faradaic constant (96,485 C mol^−1^).

$$\boldsymbol{FE=}\frac{\boldsymbol{Q}_{\boldsymbol{product}}}{\boldsymbol{Q}_{\boldsymbol{total}}}\boldsymbol{=}\frac{\boldsymbol{m}\boldsymbol{\times}\boldsymbol{n}\boldsymbol{\times}\boldsymbol{F}}{\boldsymbol{Q}_{\boldsymbol{total}}}\boldsymbol{(2)}$$

1. **Characterizations of catalysts**

**
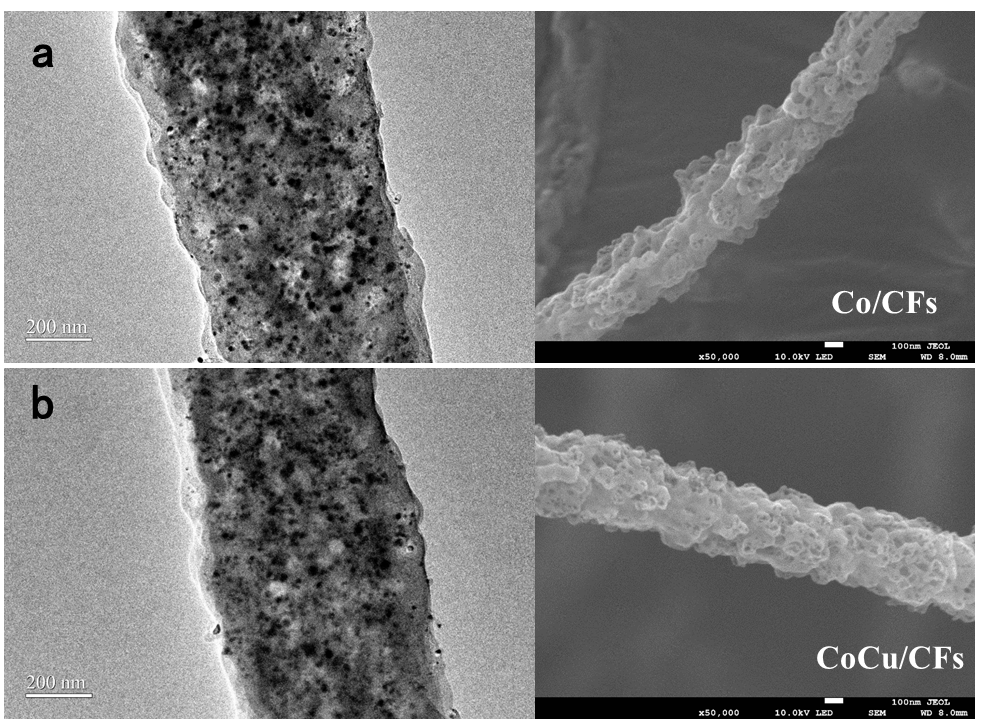
**

**Supplementary Figure 1.** TEM and SEM images of Co/CFs (a) and CoCu/CFs (b).


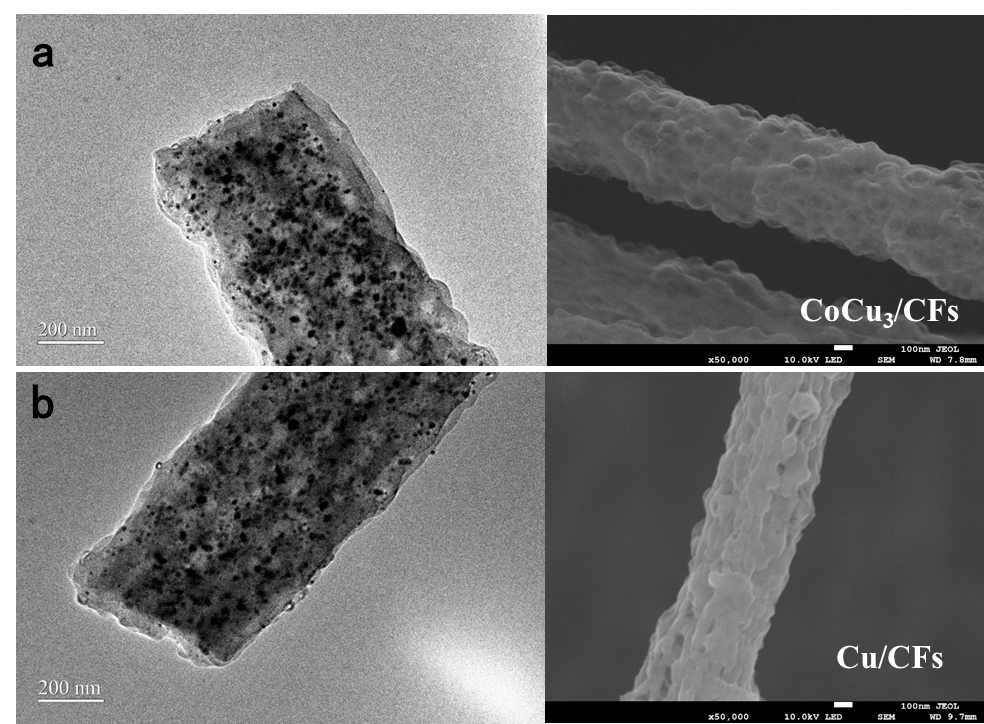


**Supplementary Figure 2.** TEM and SEM images of CoCu_3_/CFs (a) and Cu/CFs (b).


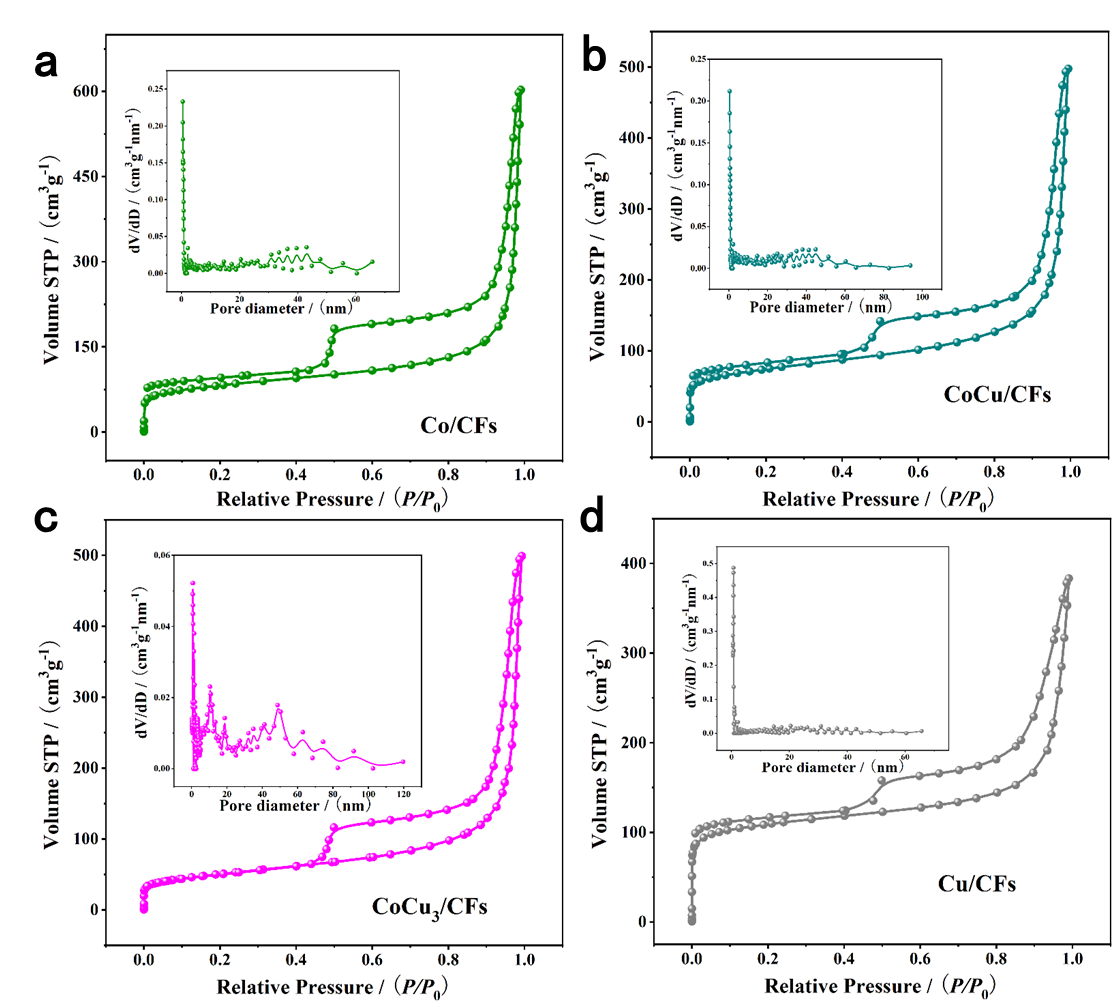


**Supplementary Figure 3.** N_2_ sorption isotherms and pore size distributions of Co/CFs (a), CoCu/CFs (b), CoCu_3_/CFs (c) and Cu/CFs (d), respectively.

**Table S1** BET data of various catalysts.

| Sample | Total pore volume / cm^3^ g^-1^ | Specific surface area / m^2^ g^-1^ |
| --- | --- | --- |
| Co/CFs | 0.7155 | 265 |
| Co_3_Cu/CFs | 0.9176 | 292 |
| CoCu/CFs | 0.7359 | 261 |
| CoCu_3_/CFs | 0.7336 | 181 |
| Cu/CFs | 0.7085 | 178 |


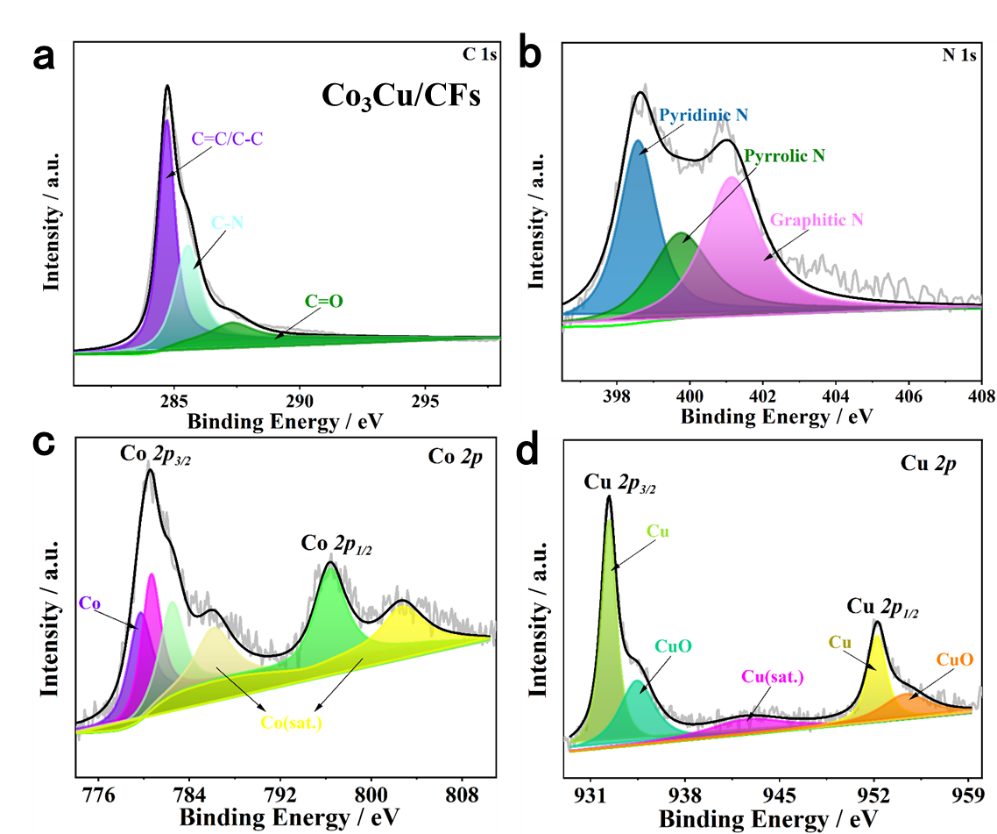


**Supplementary Figure 4.** C *1s* (a), N *1s* (b), Co *2p* (c) and Cu *2p* (d) XPS spectra of Co_3_Cu/CFs.


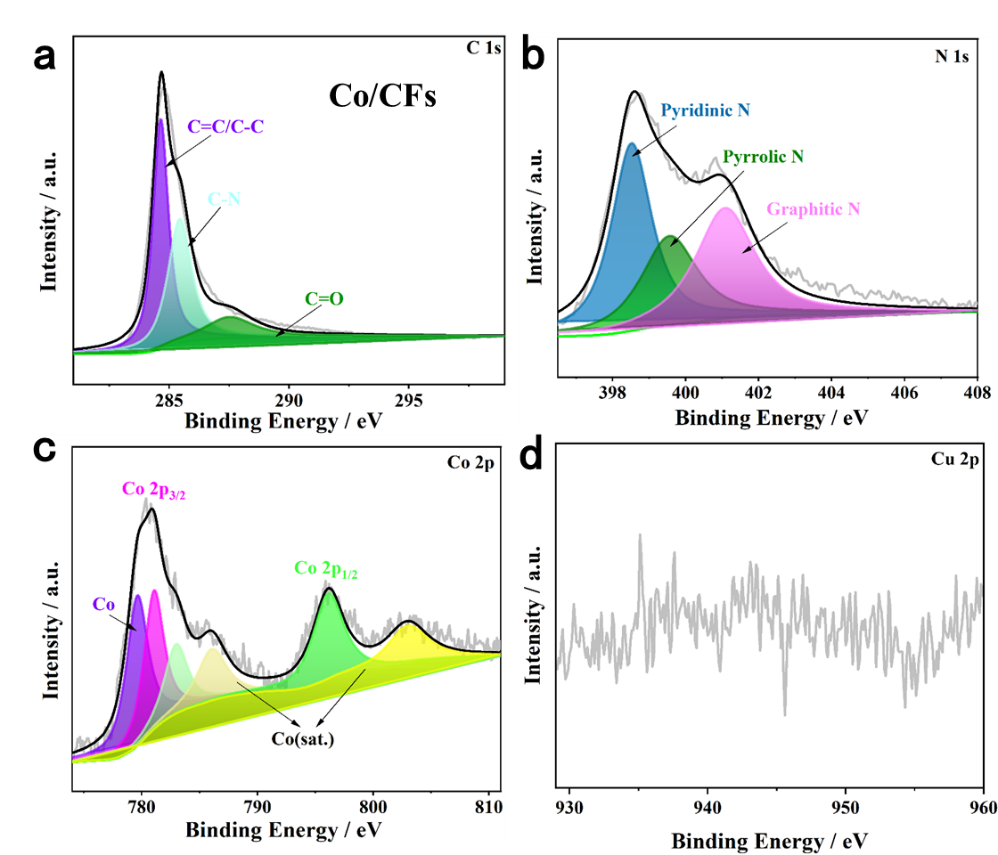


**Supplementary Figure 5.** C *1s* (a), N *1s* (b), Co *2p* (c) and Cu *2p* (d) XPS spectra of Co/CFs.


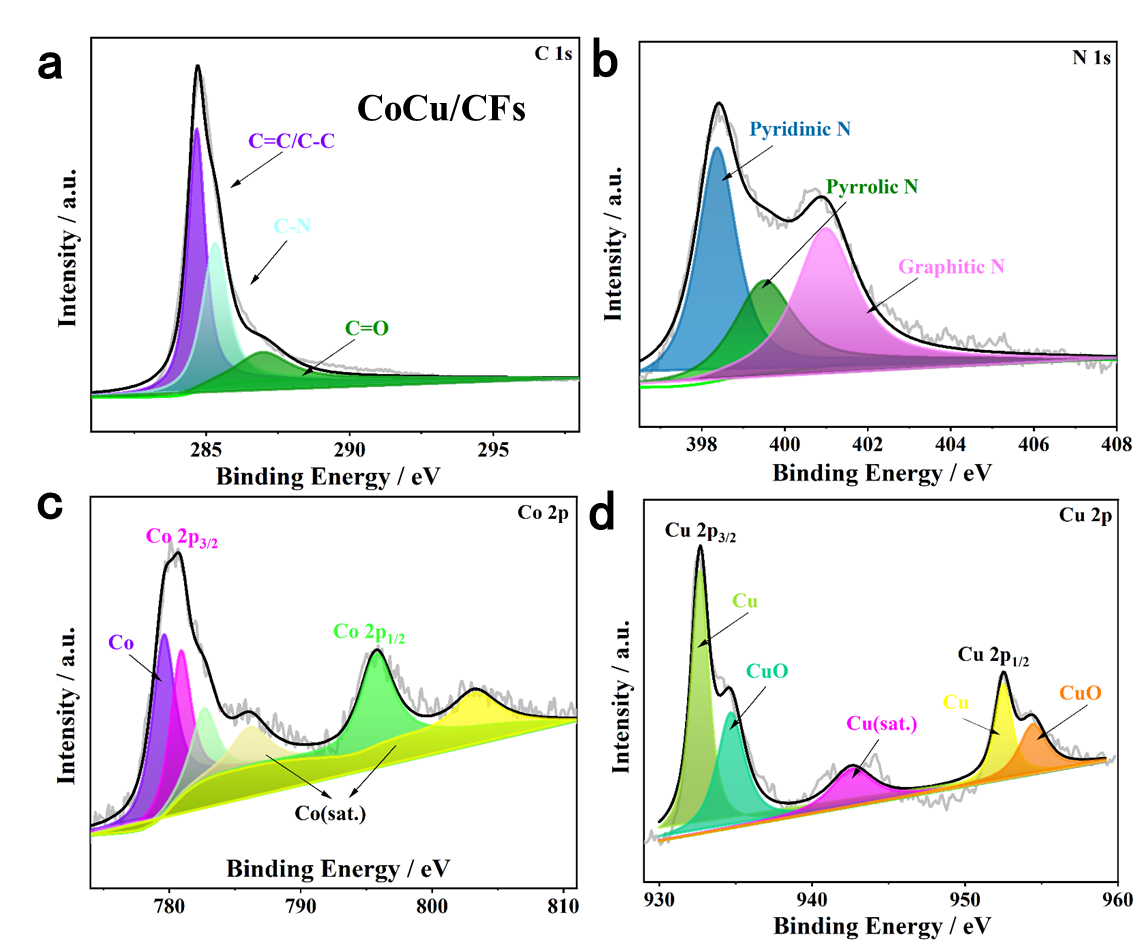


**Supplementary Figure 6.** C *1s* (a), N *1s* (b), Co *2p* (c) and Cu *2p* (d) XPS spectra of CoCu/CFs.


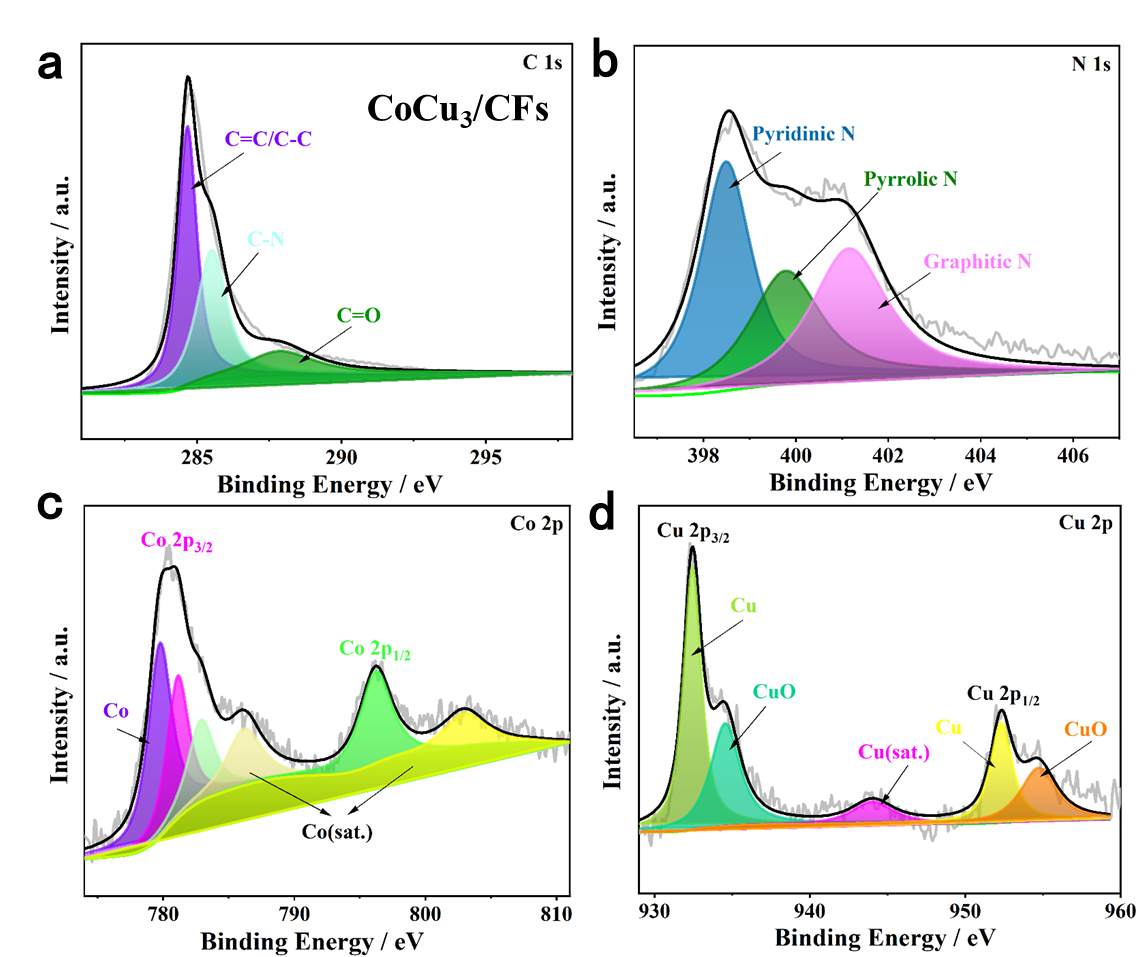


**Supplementary Figure 7.** C *1s* (a), N *1s* (b), Co *2p* (c) and Cu *2p* (d) XPS spectra of CoCu_3_/CFs.


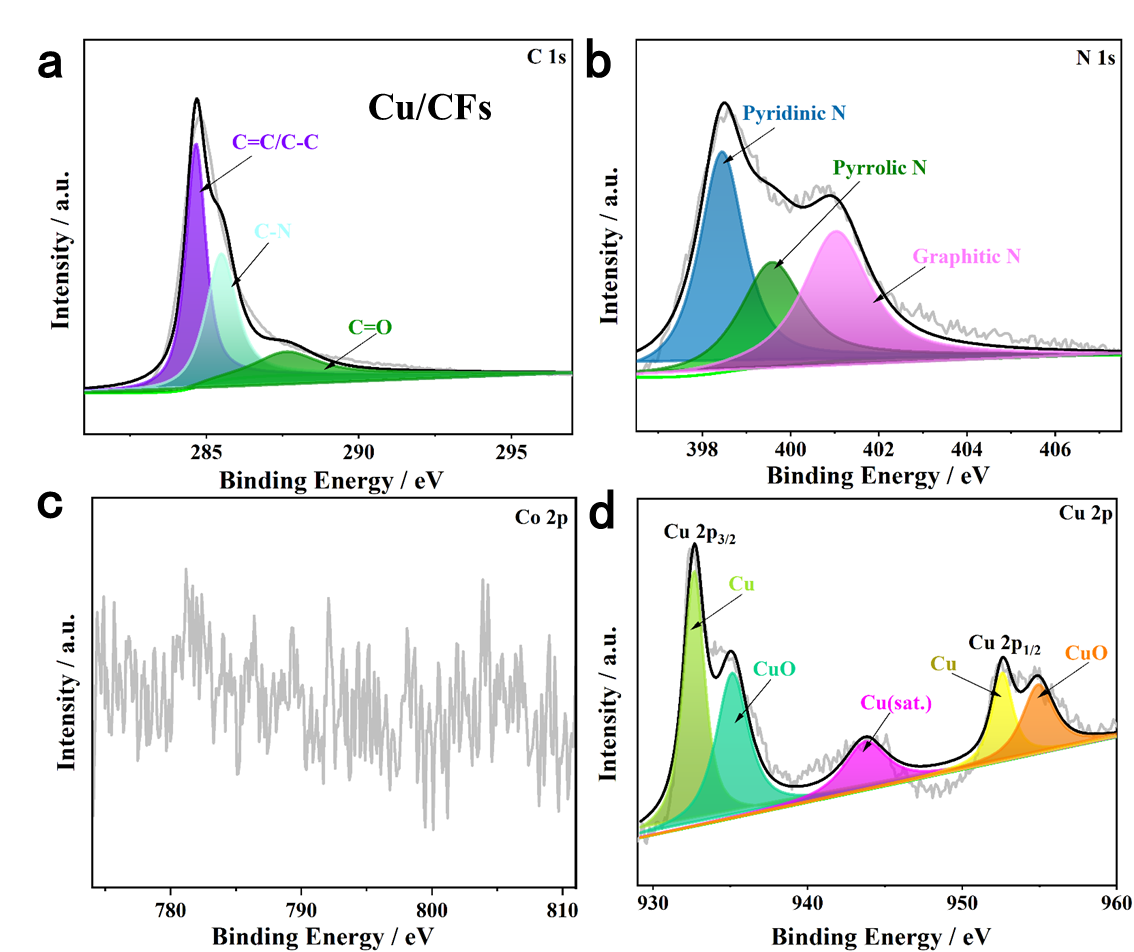


**Supplementary Figure 8**. C *1s* (a), N *1s* (b), Co *2p* (c) and Cu *2p* (d) XPS spectra of Cu/CFs.

1. **Electrochemical reduction of CO_2_ using different catalysts**

**
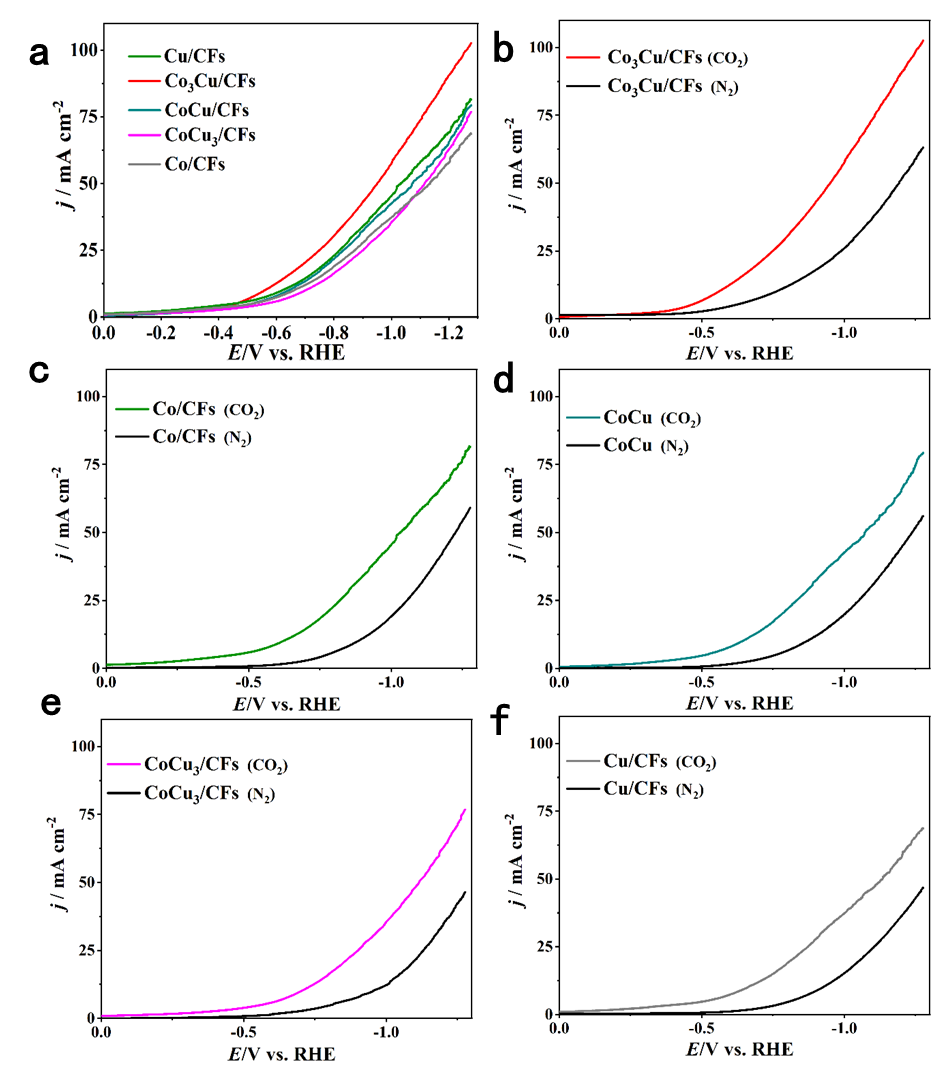
**

**Supplementary Figure 9**. LSV curves in CO_2_-saturated and N_2_-saturated 0.5 M KHCO_3_ solution in H-type cell using five catalysts.


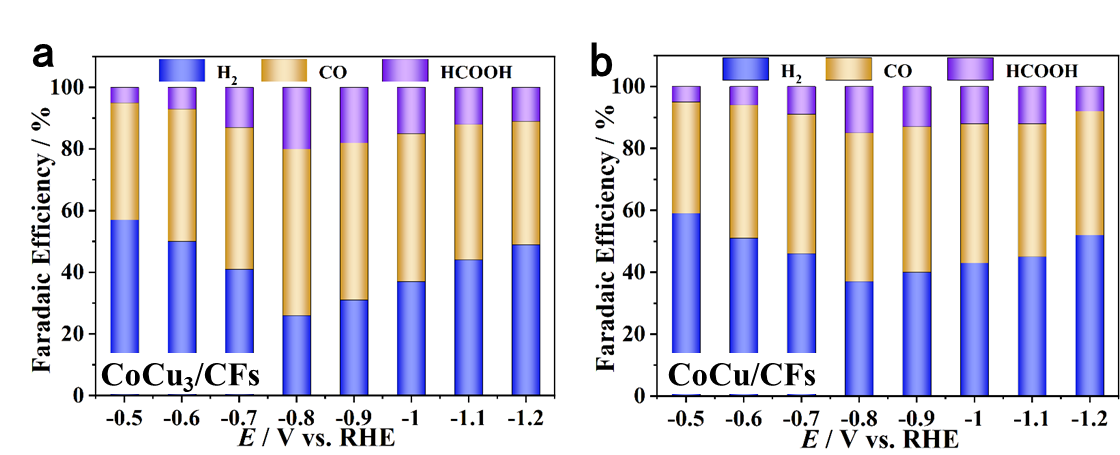


**Supplementary Figure 10.** Faradaic efficiencies of reduction products using CoCu_3_/CFs (a), CoCu/CFs (b).


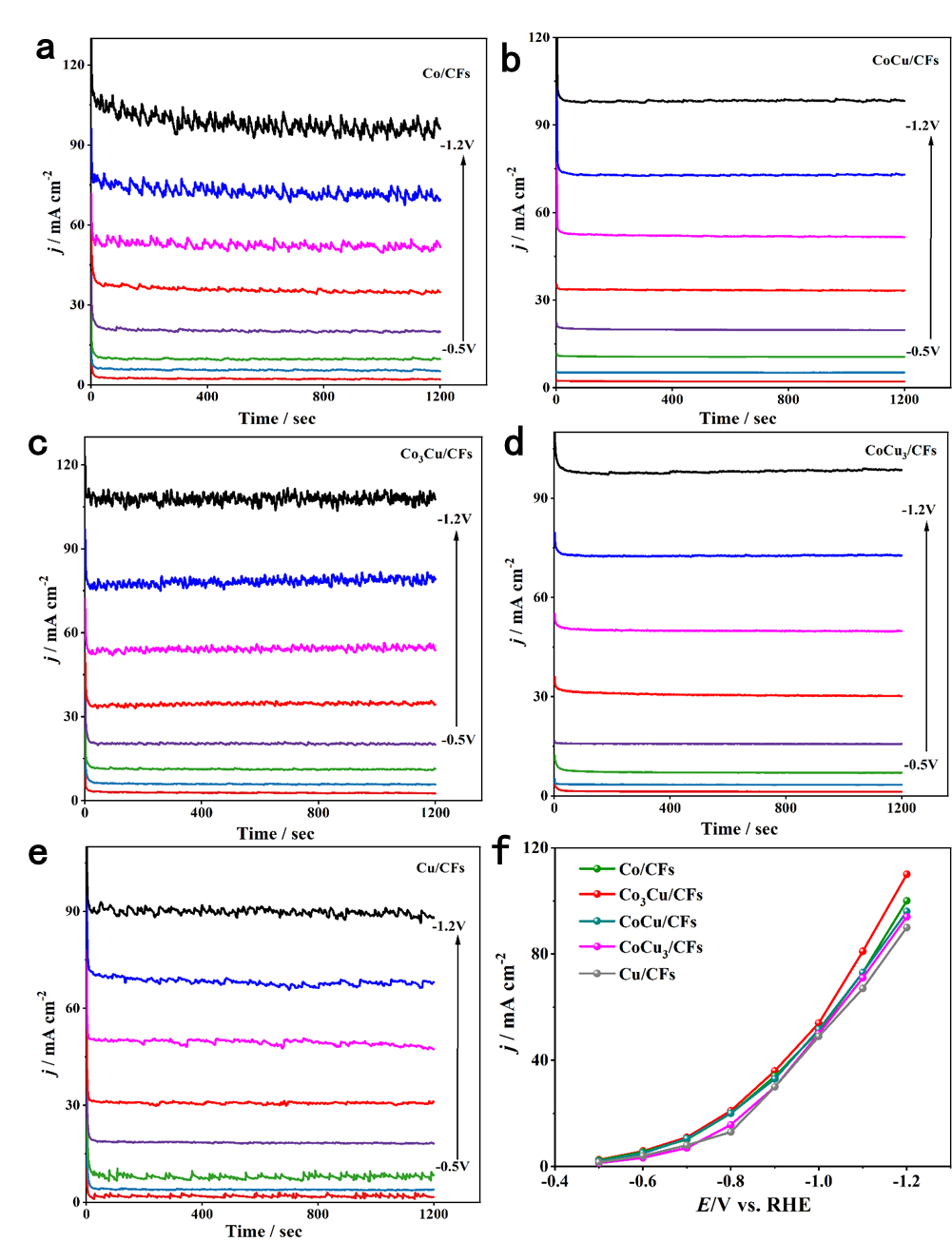


**Supplementary Figure 11.** Total current density of five samples at −0.5 V_RHE_ to −1.2 V_RHE_ cathode potentials in 0.5 M KHCO_3_ electrolyte.


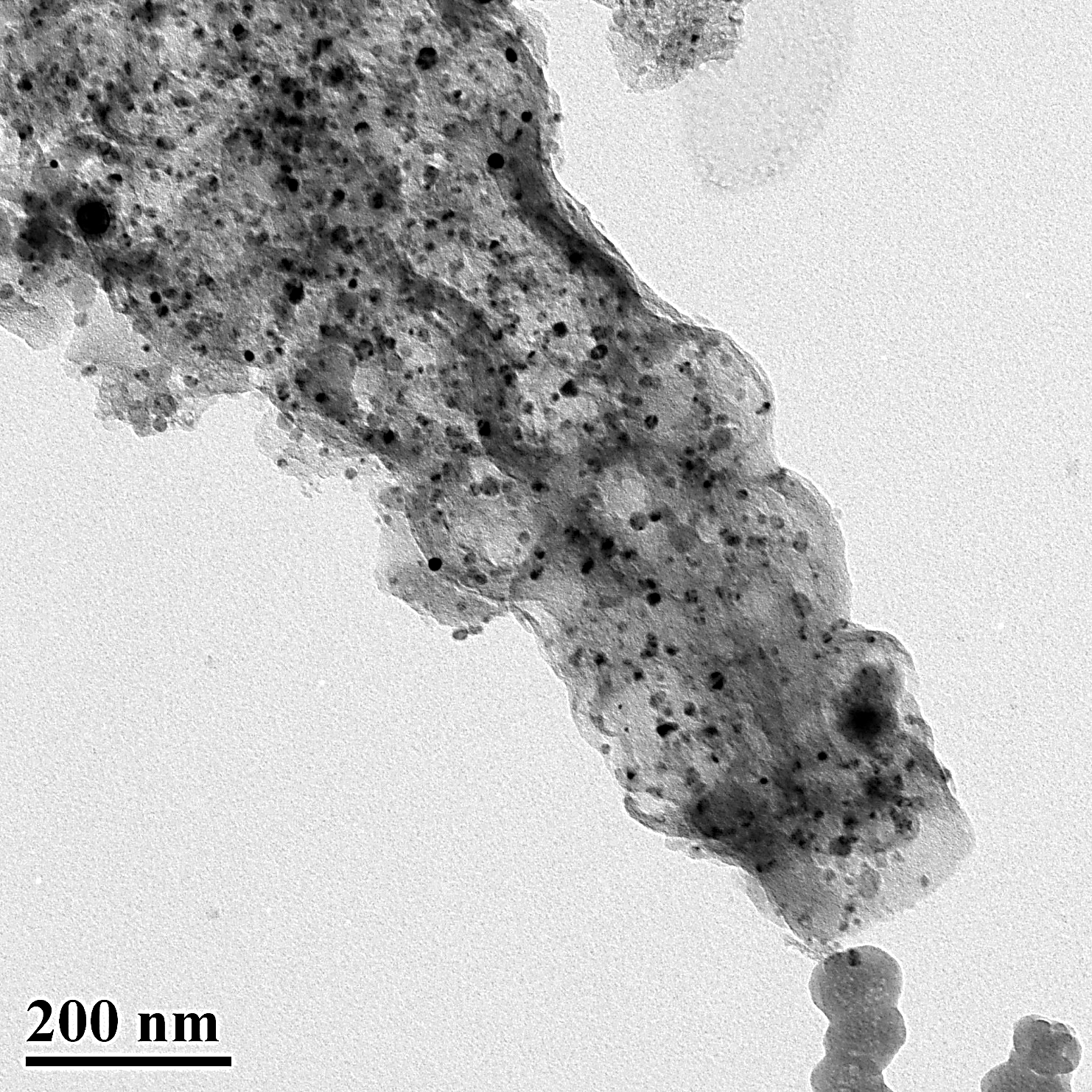


**Supplementary Figure 12**. The TEM image of Co_3_Cu/CFs after long-term electrolysis.


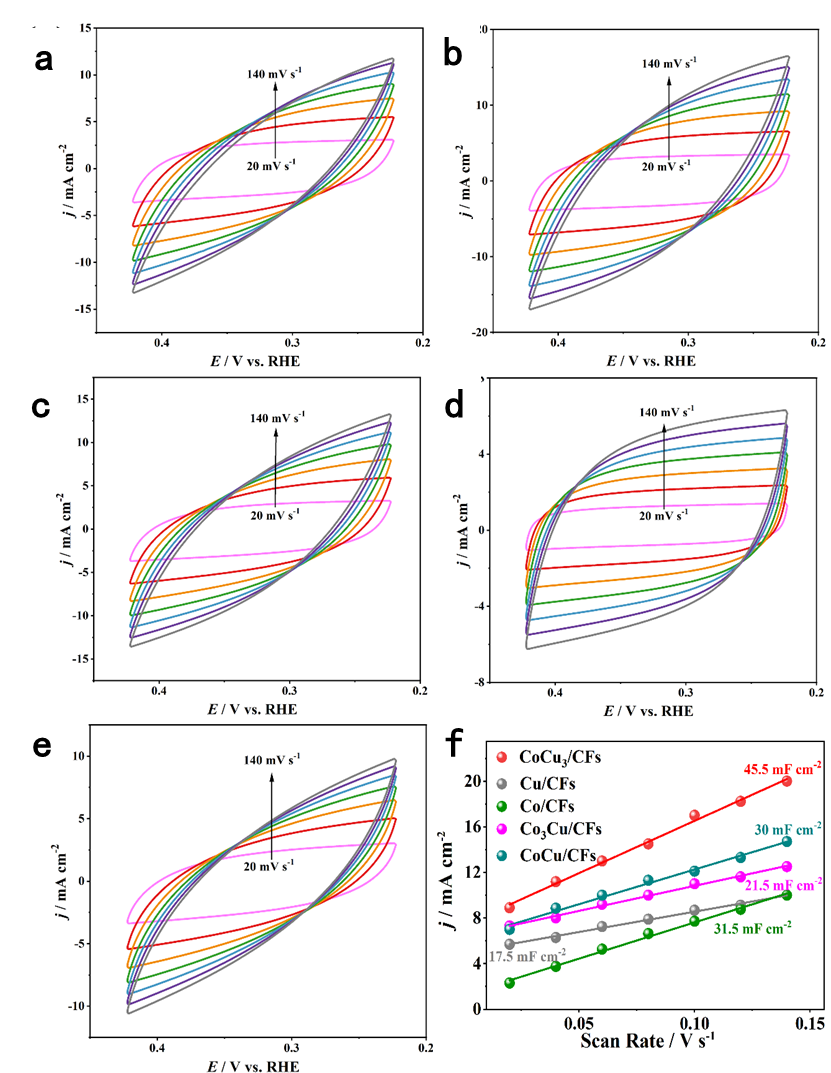


**Supplementary Figure 13**. CVs of five samples (a-e) from 0.2 to 0.4 V_RHE_ at various scan rates (0.02 to 0.14 V s^-1^); double layer capacitances of five samples (f).
